# 1127. Effectiveness of Remdesivir as Treatment for COVID-19 Positive US Veterans

**DOI:** 10.1093/ofid/ofac492.966

**Published:** 2022-12-15

**Authors:** Sujee Jeyapalina, Margaret Lundquist, Guo Wei, Gregory Stoddard, Jay Agarwal

**Affiliations:** University of Utah, SALT LAKE CITY, Utah; Department of Veterans Affairs, SALT LAKE CITY, Utah; University of Utah, SALT LAKE CITY, Utah; University of Utah, SALT LAKE CITY, Utah; University of Utah, SALT LAKE CITY, Utah

## Abstract

**Background:**

The COVID 19 disease has claimed over 6.3 million lives, globally. Despite such high casualties, the treatment options are limited. Although the FDA issued emergency use authorizations for oral antivirals to treat mild-to-moderate COVID 19 disease, intravenous Remdesivir treatment remains the only fully FDA-approved antiviral. However, many early studies questioned its efficacy. Accordingly, the WHO initially recommended against its use in COVID 19 positive patients. Based on the newly emerging data, as of 22 April 2022, WHO suggests that Remdesivir can be effectively used in mild or moderate COVID 19 cases. This retrospective cohort data analysis was undertaken to evaluate and clarify the effectiveness of Remdesivir use in older US veterans.

**Methods:**

The deidentified veterans' data were accessed from the VA COVID 19 Shared Data Resources with local ethical approvals. Propensity matched cohorts with and without Remdesivir treatment were analyzed using Cox regression models, constructed in a way to avoid immortal time and calendar time biases. Limited to hospitalized veterans, patients were followed for 60 days to the outcomes of mechanical ventilation (MV) and death in separate models. The cohort was also limited to those who received low flow without high flow oxygen and a combination of low and high flow oxygen in another set of models.

**Results:**

A total of 3,372 veterans were included in this study who were hospitalized between 01 January to 31 December 2021 for COVID 19 disease. Of those, 1,686 received Remdesivir treatment, while their matches never received it. After propensity score matching that included demography, vaccination status, comorbidities, medication use, lab tests, Remdesivir recipients and controls were similar in age (66.8±14.1 vs. 67.0±13.8 years). Relative risk reductions (1-HR), 53% for MV, and 42% for death (Fig. 1) were observed with low flow oxygen and Remdesivir therapy. In veterans who received high and low flow oxygen, although there was a significant 18% reduction in risk for death, progression to MV was not significant (P=0.22).

**Figure 1:**
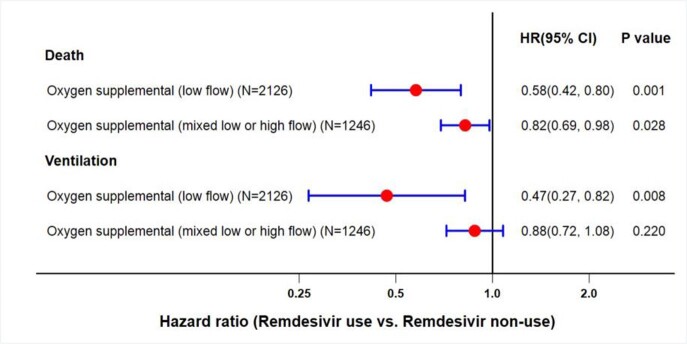
A forest plot showing the hazard ratios (HRs) for death and mechanical ventilator use in hospitalized US veterans with COVID-19 positivity between 01st of January to 31st of December 2021.

**Conclusion:**

The data showed significant risk reductions of disease progression to MV/death when Remdesivir was used in COVID 19 positive patients with low supplementary oxygen flow, supporting the current NIH recommendation.

**Disclosures:**

**Sujee Jeyapalina, PhD**, Dr. Jeyapalina received funding from Gilead Sciences, Inc. for COVID-19 data research: Grant/Research Support **Gregory Stoddard, Mstat**, Greg Stoddard received funding from Gilead Sciences, Inc. for COVID-19 data research: Grant/Research Support **Jay Agarwal, MD**, Dr. Agarwal received funding from Gilead Sciences, Inc. for COVID-19 data research: Grant/Research Support.

